# Direct healthcare costs associated with device assessed and self-reported physical activity: results from a cross-sectional population-based study

**DOI:** 10.1186/s12889-018-5906-7

**Published:** 2018-08-03

**Authors:** Florian M. Karl, Maximilian Tremmel, Agnes Luzak, Holger Schulz, Annette Peters, Christa Meisinger, Rolf Holle, Michael Laxy

**Affiliations:** 1 Institute of Health Economics and Health Care Management, Helmholtz Zentrum München, German Research Center for Environmental Health, Ingolstädter Landstr.1, 85764 Neuherberg, Germany; 2 Institute for Medical Information Processing, Biometrics and Epidemiology (IBE), Ludwig-Maximilians-University Munich, Marchioninistr. 15, 81377 Munich, Germany; 3 Institute of Epidemiology I, Helmholtz Zentrum München, German Research Center for Environmental Health, Ingolstädter Landstr.1, 85764 Neuherberg, Germany; 4 Institute of Epidemiology II, Helmholtz Zentrum München, German Research Center for Environmental Health, Ingolstädter Landstr.1, 85764 Neuherberg, Germany

**Keywords:** Physical activity, Direct healthcare costs, Accelerometer, Questionnaire, KORA, Cross-sectional

## Abstract

**Background:**

Physical inactivity (PIA) is an important risk factor for many chronic conditions and therefore might increase healthcare utilization and costs. This study aimed to analyze the association of PIA using device assessed and self-reported physical activity (PA) data with direct healthcare costs.

**Methods:**

Cross-sectional data was retrieved from the population based KORA FF4 study (Cooperative Health Research in the Region of Augsburg) that was conducted in southern Germany from 2013 to 2014 (*n* = 2279). Self-reported PA was assessed with two questions regarding sports related PA in summer and winter and categorized into “high activity”, “moderate activity”, “low activity” and “no activity”. In a subsample (*n* = 477), PA was assessed with accelerometers and participants were categorized into activity quartiles (“very high”, “high”, “low” and “very low”) according to their mean minutes per day spent in light intensity, or in moderate-vigorous PA (MVPA). Self-reported healthcare utilization was used to estimate direct healthcare costs. We regressed direct healthcare costs on PA using a two-part gamma regression, adjusted for age, sex and socio-demographic variables. Additional models, including and excluding potential additional confounders and effect mediators were used to check the robustness of the results.

**Results:**

Annual direct healthcare costs of individuals who reported no sports PA did not differ from those who reported high sports PA [+€189, 95% CI: -188, 598]. In the subsample with accelerometer data, participants with very low MVPA had significantly higher annual costs than participants with very high MVPA [+€986, 95% CI: 15, 1982].

**Conclusion:**

Device assessed but not self-reported PIA was associated with higher direct healthcare costs. The magnitude and significance of the association depended on the choice of covariates in the regression models. Larger studies with device assessed PA and longitudinal design are needed to be able to better quantify the impact of PIA on direct healthcare costs.

**Electronic supplementary material:**

The online version of this article (10.1186/s12889-018-5906-7) contains supplementary material, which is available to authorized users.

## Background

Physical inactivity (PIA) is prevalent in 31% of the world’s population, and has been identified as the fourth leading risk factor for global mortality causing 6% of deaths worldwide [1–3].

Valid estimates on the financial burden of PIA are rare due to the complexity of measuring PIA and healthcare costs in large-scale studies [4]. However, quantification of the financial burden of PIA will provide evidence for Public Health policy makers to prioritize efforts to increase PA on a population level.

Previous studies have estimated the percentage of direct healthcare costs associated with PIA using a population-attributable fraction approach that combines risk, prevalence, and aggregate cost estimates from various sources [5–10]. For example, Allender et al. [5] used information from the WHO global burden of disease report of 2002 and concluded that the costs of PIA due to mortality and morbidity to the National Health Service in the UK is £1.06 billion annually.

Another approach to calculate direct healthcare costs is to use individual data linked to healthcare expenditure data. This approach is called *bottom-up approach* and is overcoming many of the limitations associated with estimates calculated using a population-attributable fraction approach [11, 12]. Carlson et al. [11] used physical activity (PA) data from the National Health Interview Survey merged with data from the Medical Expenditure Panel Survey and used a four-part econometric model to calculate mean differences in direct healthcare costs between inactive and active adults. In this study, inactive subjects had $1437 higher overall direct healthcare costs as compared to active.

PA is an ambiguous concept as its forms and intensity levels vary substantially. Therefore, the measurement through self-report is highly vulnerable to biases in perception and reporting that can lead to under- or overestimation of PA [13]. Alternatively, PA can be measured using devises such as accelerometers. These devices have gained popularity due to their ability to capture large amounts of information on individual activity profiles in population-based studies. Among several strengths of accelerometers, a limitation is that they do not provide any contextual information (i.e. type of sport, additional equipment used, for example, weights used for weight training) [14]. Neither the device assessed, nor the self-reported assessment method alone gives a valid and comprehensive picture of people’s PA level.

The aim of this study is to analyze the association between PIA and direct healthcare costs using both, self-report and device assessed measures of PA in a large population-based cross-sectional study.

## Methods

### Study population and sampling

We used cross-sectional data of the population based KORA FF4 study (Cooperative Health Research in the Region of Augsburg) that was conducted in southern Germany from 2013 to 2014. KORA FF4 included 2279 participants and is the second follow-up study to the population based KORA S4 study that was conducted from 1999 to 2001 (*n* = 4261). KORA is a regional research platform for population-based surveys and subsequent follow-up studies in the fields of epidemiology, health economics, and healthcare research. A subsample from the FF4 study (those aged between 48 to 68 years) was designated to participate in the “Lung health & physical activity” examination and was asked to take part in the PA assessment by an accelerometer, of which 477 provided valid measurements and were included in this analysis.

Detailed information about the study design, sampling methods, response rates and dropouts of the FF4 study and the accelerometer assessment in the subsample are described in Kowall et al. and Luzak et al. [15].

### Assessment of physical activity

#### Questionnaire

Sports related PA was assessed by two questions: “How often do you exercise during winter?” and, “How often do you exercise during summer?”. The possible responses for each question were “regularly more than two hours per week”, “regularly one to two hours per week”, “less than one hour per week” and “no activity”. The two responses for summer and winter were then combined [see Additional file 1] to a variable that was categorized with (1) “high activity”, (2) “moderate activity”, (3) “low activity” and (4) “no activity”. This question has been validated by using a physical activity diary as a comparison [16].

#### Accelerometer

The participants received the accelerometer and instructions on its use at the study centre. The measurement of PA was obtained from ActiGraph GT3X (Pensacola, Florida) accelerometers with the use of the ActiLife software (version 6.11.2, firmware 4.4.0). The accelerations were sampled at 30 Hz, stored in 1 s epochs and resampled in 1 minute epochs for further data analysis with data filtering set to normal as recommended by ActiGraph. The accelerometer was attached to an elastic belt and worn at the hip side of the dominant hand. Participants were asked to wear the accelerometer for 7 days from getting up to going to sleep and to report non-wear time. A non-wear time algorithm, based on the NHANES algorithm [17], was applied to assess non-wear time in the accelerometer data. Differences between the non-wear time algorithm applied to the accelerometer data and the non-wear time reported in the diary that were > 60 min (if the non-wear time was reported in the diary) or > 120 min (if non-wear time was indicated by the accelerometer) led to exclusion of this day. Further exclusion criteria were non-wear time during reported exercise in the diary > 2 h, missing information on day length, and wear time < 10 h/day. Detailed exclusion criteria have been previously published [18]. Valid measurements included at least three valid weekdays and one valid weekend day [18]. We used the uniaxial counts (counts/min) on the vertical axis for the deduction of the activity levels. The cut-offs were based on those used by Freedson et al. [19]: a light intensity activity level for > 100 to ≤1951 cpm and a moderate to vigorous activity level (MVPA) for > 1951 cpm. The obtained minutes spent in light intensity PA or MVPA were summed and averaged over the participants recording period, resulting in average minutes/day spent in light intensity PA and MVPA for each participant. According to their mean minutes per day for light intensity PA and separately for MVPA, male and female participants were categorized into four sex-specific activity quartiles (very low, low, high, very high). We excluded one individual with extreme body weight (BMI > 60 kg/m^2^) and one with an extreme value of more than 200 min/day in MVPA. More detailed information about the PA assessment with accelerometer in the KORA FF4 population has been described elsewhere [18].

Steps per day as a global indicator for the overall volume of PA were measured and participants were categorized into being active or inactive with reaching the threshold of 10,000 steps per day [20, 21]. According to the WHO PA recommendation, we additionally used the threshold of ≥150 min/week of MVPA spent in at least 10-min bouts as another overall measure of physical activity [11].

### Assessment of direct healthcare costs

Participants were asked to report the type and number of outpatient physician and ambulatory hospital visits in the previous 3 months [22], the number of inpatient hospital days and days in rehabilitation in the last 12 months, and the use of pharmaceuticals in the previous 7 days (assessed via the information on name, pharmaceutical identification number). All utilization (physician visits, ambulatory hospital visits, and drugs), if not already reported over one-year (inpatient hospital visits and rehabilitation), were extrapolated to a one-year period.

To calculate direct healthcare costs, the frequency of service use was multiplied by the unit costs provided by Bock et al. [23] and all prices refer to the year 2013 [see Additional file 2]. For this monetary valuation of health services, national unit costs were applied as recommended by the Working Group Methods in Health Economic Evaluation (AG MEG) [24]. As the reason for hospitalization was not available, hospital days were valued using mean costs per day as suggested by the AG MEG. All unit costs [see Additional file 2] were updated to the costing year 2013. For our analysis, we included costs of 15 different physicians (GPs and specialists). The extrapolated utilization of each physician over 1 year was then multiplied by the unit costs specifically for each type of physician. The number of ambulatory hospital stays, inpatient hospital stays and rehabilitation over a one-year period were also multiplied with the corresponding unit costs. Drug utilization was calculated with the defined daily dose (DDD) calculation and the calculation was limited to prescribed drugs only. The cost of medication was estimated using the pharmacy retail prices from the Scientific Institute of the AOK healthcare insurance (WIdO) and the price index calculator of the Federal Statistical Office. Total direct medical costs were then calculated as the sum of the above-mentioned cost categories over a one-year period. A more detailed description of the calculation of the direct costs in the KORA cohort has been reported elsewhere [25].

### Covariates

In our study, all information (except accelerometer data) was either gathered by trained staff during a standardized face-to-face interview or by self-report questionnaire. We defined education by primary (< 9 years), secondary (10 years), and tertiary (> 10 years) education and assessed the equivalence income as measures of socio economic status [26]. Furthermore, we differentiated among never, former and active smokers. Reported daily alcohol consumption was transformed into a binary variable indicating elevated alcohol consumption as > 12 g/day for women and > 24 g/day for men [27]. For self-reported information on history of myocardial infarction (MI), stroke, cancer, diabetes and asthma binary variables (yes-no) were coded. Problems in walking were assessed with a question from the EQ-5D-5 L questionnaire (item mobility) and all subjects who reported moderate, or severe problems with walking or who reported to be unable to walk about were grouped as subjects who had problems with walking.

### Statistical analysis

Cost data typically present challenges because of their skewness and a high percentage of zeros. We decided to use a two-part gamma model since zero-costs were reported by 17.2% of the participants and the cost distribution was highly right-skewed. Resulting mean costs were estimated through recycled predictions from 500 bootstrapping replications. Extreme values (i.e. > 99th percentile) were replaced by the value of the 99th percentile in order to avoid outliers due to recall bias.

In order to have a better understanding of the association between PIA and direct healthcare costs, an overview of potential confounders and mediators is represented in Additional file 3. Analyses were performed with two sets of covariates. In *Model A* we adjusted for age, age^2^, sex, education and income. Underlying diseases might have influenced the participants’ PA and healthcare costs. Thus, they need to be considered as possible confounders to the association between PA and direct healthcare costs. Therefore, we adjusted *Model B* for chronic diseases (diabetes, asthma, stroke, MI and cancer). Additionally, *Model B* was adjusted for known risk factors (smoking status and alcohol consumption). We excluded participants with chronic diseases (i.e. diabetes, asthma, stroke, MI, and cancer) in a third model (*Model C*) in order to investigate the association of PA and direct healthcare costs in a supposedly “healthy” population. In *Model D*, we adjusted for the variables from *Model A* but excluded all subjects who reported problems with walking to address reverse causation.

The mean wear time in individuals with very high MVPA equaled 923 min ± 65 min and 911 min ± 63 min in individuals with very low MVPA. Overall, there was no significant difference in wear time between the four MVPA subgroups (*p*-value = 0.44). In participants with very high light intensity PA and very low light intensity PA the mean wear time of the device was 940 min ± 60 min and 903 min ± 69 min respectively. Overall, the mean wear time differed significantly among the four light intensity PA subgroups (p-value < 0.001). Therefore, *Model E* was similar to *Model A,* but additionally adjusted for wear time and therefore only applied to the subset. Finally, *Model F* was only applied to the MVPA and light intensity PA domains of the subset as it was also similar to *Model A* but in case of MVPA additionally adjusted for light intensity PA and vice versa. Statistical analyses were performed using SAS software (SAS Institute Inc., Cary, NC, USA, Version 9.2).

## Results

### Description of the study population

We included *n* = 2279 subjects in our study with a mean age of 60 ± 12 years of which 52% were female and according to their self-reported PA status, the sample was distributed the following: 26% with high activity, 31% with moderate, 14% with low and 28% with no sports activity (Table 1).

**Table 1:** Description of the total study sample of KORA FF4 and self-reported physical activity groups with means or frequencies (standard deviations or percentages)

Variable		Physical activity during sports			
		*High activity*	*Moderate activity*	*Low activity*	*No activity*
N	2279	596 (26.2)	709 (31.1)	329 (14.4)	645 (28.3)
Age	60.2 (12.3)	59.4 (11.5)	59.1 (11.9)	59.3 (12.1)	62.9 (13.2)
Sex					
*Female*	1177 (51.6)	284 (47.7)	410 (57.8)	174 (52.9)	309 (47.91)
Education					
*Primary*	1110 (48.8)	224 (37.7)	327 (46.12)	186 (56.7)	373 (58.0)
*Secondary*	585 (25.7)	172 (29.0)	200 (28.2)	79 (24.1)	134 (20.8)
*Tertiary*	579 (25.4)	198 (33.3)	182 (25.7)	63 (19.2)	136 (21.2)
Equivalence Income	1460.1 (679.7)	1576.3 (692.1)	1533.1 (668.2)	1386.9 (712.6)	1307.5 (629.9)
Diseases	671 (29.4)	164 (27.5)	183 (25.8)	108 (32.8)	216 (33.5)
MI	79 (3.5)	19 (3.2)	16 (2.3)	12 (3.7)	32 (5.0)
Stroke	63 (2.8)	13 (2.2)	13 (1.8)	7 (2.1)	30 (4.7)
Cancer	257 (11.3)	71 (11.9)	64 (9.0)	40 (12.2)	82 (12.7)
Diabetes	233 (10.2)	41 (6.9)	50 (7.1)	41 (12.5)	101 (15.7)
Asthma	201 (8.8)	53 (8.91)	64 (9.0)	26 (7.9)	58 (9.0)
Smoking					
*Never*	947 (41.5)	230 (38.6)	331 (46.7)	126 (38.3)	260 (40.3)
*Former*	980 (43.0)	285 (47.8)	284 (40.1)	146 (44.4)	265 (41.1)
*Yes*	352 (15.5)	81 (13.6)	94 (13.3)	57 (17.3)	120 (18.6)
Elevated alcohol consumption	675 (29.6)	199 (33.5)	214 (30.2)	90 (27.4)	172 (26.7)
Problems with walking	199 (8.8)	24 (4.1)	44 (6.2)	35 (10.7)	96 (15.1)

In the subsample (*n* = 477) 53% were female, the mean age was 58 ± 6 years and subjects were equally distributed into quartiles concerning their status in MVPA or in light intensity PA (Table 2). Compared to the total sample, subjects in the subsample had comparable characteristics concerning education status, smoking status and alcohol consumption and underlying disease status.

**Table 2:** Description of the subsample of KORA FF4 and device assessed physical activity groups with means or frequencies (standard deviations or percentages)

Variable		MVPA (Quartiles)				Light intensity physical activity (Quartiles)				Step counts per day^a^		WHO threshold^b^	
		*Very high*	*High*	*Low*	*Very low*	*Very high*	*High*	*Low*	*Very low*	Active	Inactive	Active	Inactive
N	477	119 (25.0)	118 (24.7)	117 (24.5)	123 (25.8)	118 (24.7)	120 (25.2)	119 (25.0)	120 (25.2)	98 (20.5)	379 (79.5)	66	411
Age	57.8 (5.5)	57.0 (5.2)	56.9 (5.4)	57.3 (5.7)	60.1 (5.4)	57.1 (4.9)	57.9 (5.5)	57.9 (5.8)	58.4 (5.8)	56.7 (5.2)	58.2 (5.6)	57.4 (5.4)	57.9 (5.6)
Sex													
*Female*	253 (53.1)	63 (52.9)	62 (52.5)	64 (54.7)	64 (52.0)	62 (52.5)	64 (53.3)	63 (52.9)	64 (53.3)	51 (48.0)	177 (46.7)	30 (45.7)	223 (54.3)
Education													
*Primary*	220 (46.1)	48 (40.3)	50 (42.4)	52 (44.4)	70 (56.9)	65 (55.1)	60 (50.0)	49 (41.2)	46 (38.3)	43 (43.9)	177 (46.7)	19 (28.8)	201 (48.9)
*Secondary*	138 (28.9)	43 (36.1)	31 (26.3)	35 (29.9)	29 (23.6)	34 (28.8)	39 (32.5)	27 (22.7)	38 (31.7)	33 (33.7)	105 (27.7)	26 (39.4)	112 (27.3)
*Tertiary*	119 (25.0)	28 (23.5)	37 (31.4)	30 (25.6)	24 (19.5)	19 (16.1)	21 (17.5)	43 (36.1)	36 (30.0)	22 (22.5)	97 (25.6)	21 (31.8)	98 (23.8)
Equivalence Income	1610.4 (761.8)	1516.6 (674.6)	1715.6 (825.4)	1725.0 (838.5)	1491.6 (675.5)	1543.8 (805.6)	1505.4 (572.0)	1686.5 (821.0)	1702.2 (810.3)	1534.2 (689.8)	1629.8 (778.8)	1704.1 (752.9)	1595.6 (763.2)
Diseases	116 (24.3)	25 (21.0)	27 (22.3)	32 (27.4)	32 (26.0)	19 (16.1)	33 (27.5)	30 (25.2)	34 (28.3)	19 (19.4)	97 (25.6)	13 (19.7)	103 (25.1)
MI	13 (2.7)	3 (2.5)	5 (4.2)	1 (0.9)	4 (3.3)	2 (1.7)	1 (0.83)	6 (5.0)	4 (3.3)	1 (1.0)	12 (3.2)	1 (1.5)	12 (2.9)
Stroke	6 (1.3)	1 (0.8)	–	3 (2.6)	2 (1.63)	–	2 (1.7)	3 (2.5)	1 (0.8)	1 (1.0)	5 (1.3)	1 (1.52)	5 (1.22)
Cancer	39 (8.2)	13 (10.9)	10 (8.5)	7 (6.0)	9 (7.3)	6 (5.1)	14 (11.7)	10 (8.4)	9 (7.5)	12 (12.2)	27 (7.12)	7 (10.6)	32 (7.8)
Diabetes	30 (6.3)	5 (4.2)	4 (3.4)	9 (7.7)	12 (9.8)	4 (3.4)	8 (6.7)	6 (5.0)	12 (10.0)	5 (5.1)	25 (6.6)	3 (4.6)	27 (6.6)
Asthma	47 (9.8)	7 (5.9)	12 (1.02)	15 (12.8)	13 (10.6)	10 (8.5)	11 (9.2)	12 (10.1)	14 (11.7)	5 (5.1)	42 (11.1)	4 (8.5)	43 (9.0)
Smoking													
*Never*	196 (41.1)	50 (42.0)	49 (41.5)	47 (40.2)	50 (40.7)	56 (47.5)	50 (41.7)	50 (42.0)	40 (33.3)	50 (51.0)	146 (38.5)	30 (16.3)	166 (40.4)
*Former*	207 (43.4)	49 (41.2)	53 (44.9)	53 (45.3)	52 (42.3)	45 (38.1)	58 (48.3)	47 (39.5)	57 (47.5)	32 (32.7)	175 (46.2)	30 (45.5)	177 (43.1)
*Yes*	74 (15.5)	20 (16.8)	16 (13.6)	17 (14.5)	21 (17.1)	17 (14.4)	12 (10.0)	22 (18.5)	23 (18.5)	16 (16.3)	58 (15.3)	6 (9.0)	68 (16.6)
Elevated alcohol consumption	160 (33.5)	41 (34.5)	44 (37.3)	36 (30.8)	39 (31.7)	42 (35.6)	37 (30.8)	42 (35.3)	39 (32.5)	32 (32.7)	128 (33.8)	24 (36.4)	136 (33.1)
Problems with walking	29 (6.1)	6 (5.1)	4 (3.4)	9 (7.7)	10 (8.1)	5 (4.2)	8 (6.7)	10 (8.4)	6 (5.0)	5 (5.1)	24 (6.4)	2 (3.1)	27 (6.6)

^a^ active: > 10.000 steps per day, inactive: ≤ 10.000 steps per day;

^b^ active: ≥ 150 min MVPA per week in ≥10-min bouts, inactive < 150 min MVPA per week in ≥10-min bouts

### Regression analysis with self-reported physical activity

**Fig. 1:**
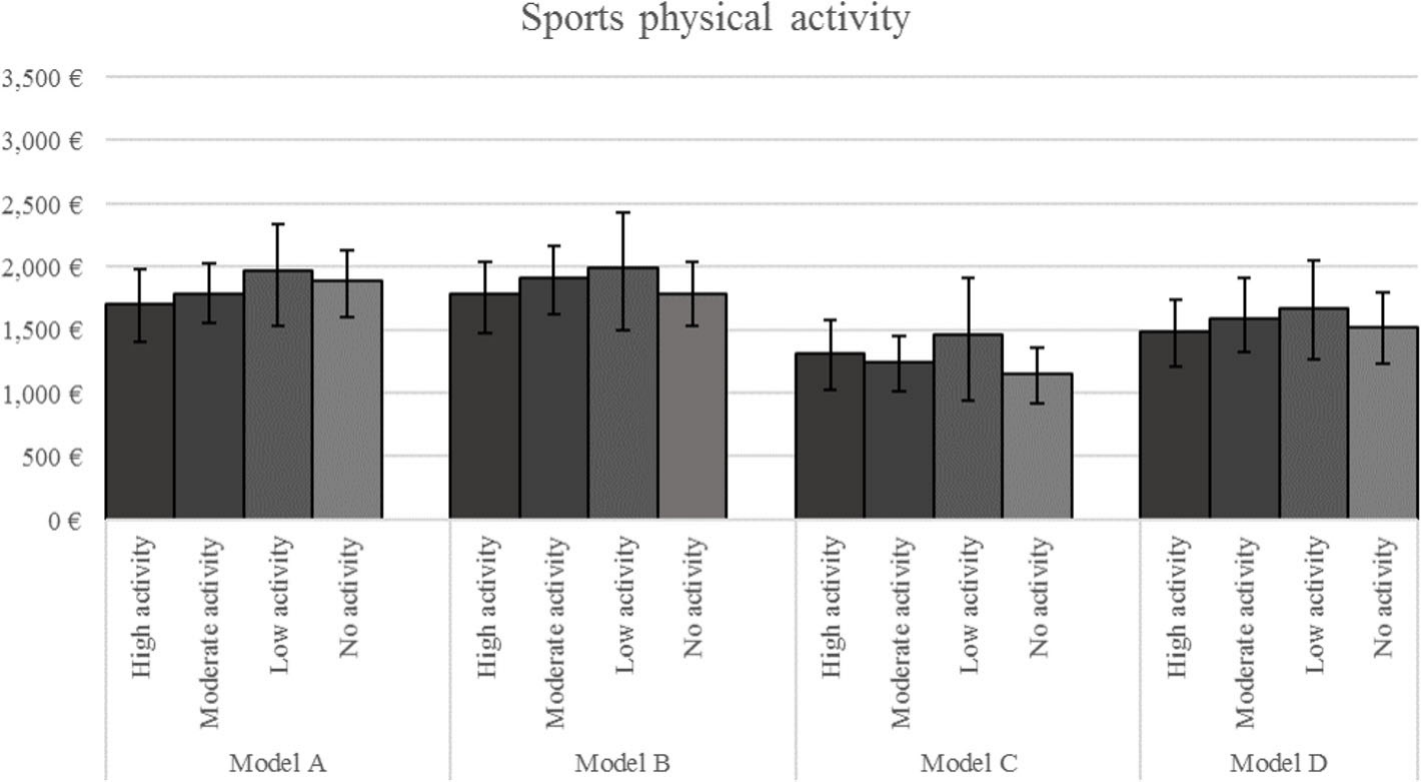
Mean overall direct healthcare costs of self-reported physical activity groups in the total sample - Results from a two-part gamma regression model

Table 3 shows the results of the two-part gamma regression model for the total sample with self-reported PA where subjects with the highest activity are the reference group and excess costs (cost differences) are reported for subjects in the lower activity groups. The estimated adjusted mean direct costs in the different PA groups are illustrated in Fig. 1.

**Table 3:** Overall direct costs (€) associated with PIA estimated with the different models in the total sample

Variable	Physical activity during sports			
	*High activity (Ref. [95% CI])*	*Moderate activity (β [95% CI])*	*Low activity (β [95% CI])*	*No activity (β [95% CI])*
Model A^a^	1700 [1423, 1996]	84 [− 346, 459.8]	275 [− 212.6–836.6]	189 [−188–598]
Model B^b^	1783 [1527, 2089]	125 [− 260, 509]	206 [−357–730]	6 [−423–391]
Model C^c^	1314 [1045, 1604]	−66 [− 431, 298]	143 [− 374–698]	−167 [− 531–197]
Model D^d^	1481 [1225, 1759]	109 [− 225, 437]	189 [− 287–679]	33 [− 365–382]

The highest level of PA was used as a reference within all four different measures of PA. Therefore, the values in the respective columns (*Ref.)* represent a mean estimate whereas the values in the other columns (*β*) represent coefficients

^a^Model A: adjusted for age, age^2^, sex, education and equivalence income;

^b^Model B: adjusted for variables in Model A plus diabetes, asthma, MI, stroke, cancer, smoking status and alcohol consumption;

^c^Model C: adjusted for variables in Model A & exclusion of participants with prevalent chronic diseases mentioned in Model B (*n* = 1608);

^d^Model D: adjusted for variables in Model A & exclusion of participants with problems with walking (*n* = 2059);

** *p* < 0.05, * trend with *p* < 0.10

When adjusted for age, age^2^, sex, education and equivalence income (*Model A*) the excess direct healthcare costs were insignificantly higher [€189, 95% CI: -188, 598] in reference to the subjects with high activity in sports [€1700, 95% CI: 1423, 1996]. In Model B, the difference between individuals with high and no PA was smaller and also insignificant. In the analysis with *Model C*, where subjects with stroke, MI, diabetes, cancer, and asthma were excluded, inactive participants had the lowest overall costs, but this difference was also insignificant [€-167, 95% CI: -531, 197].

### Regression analysis with accelerometer data

**Fig. 2:**
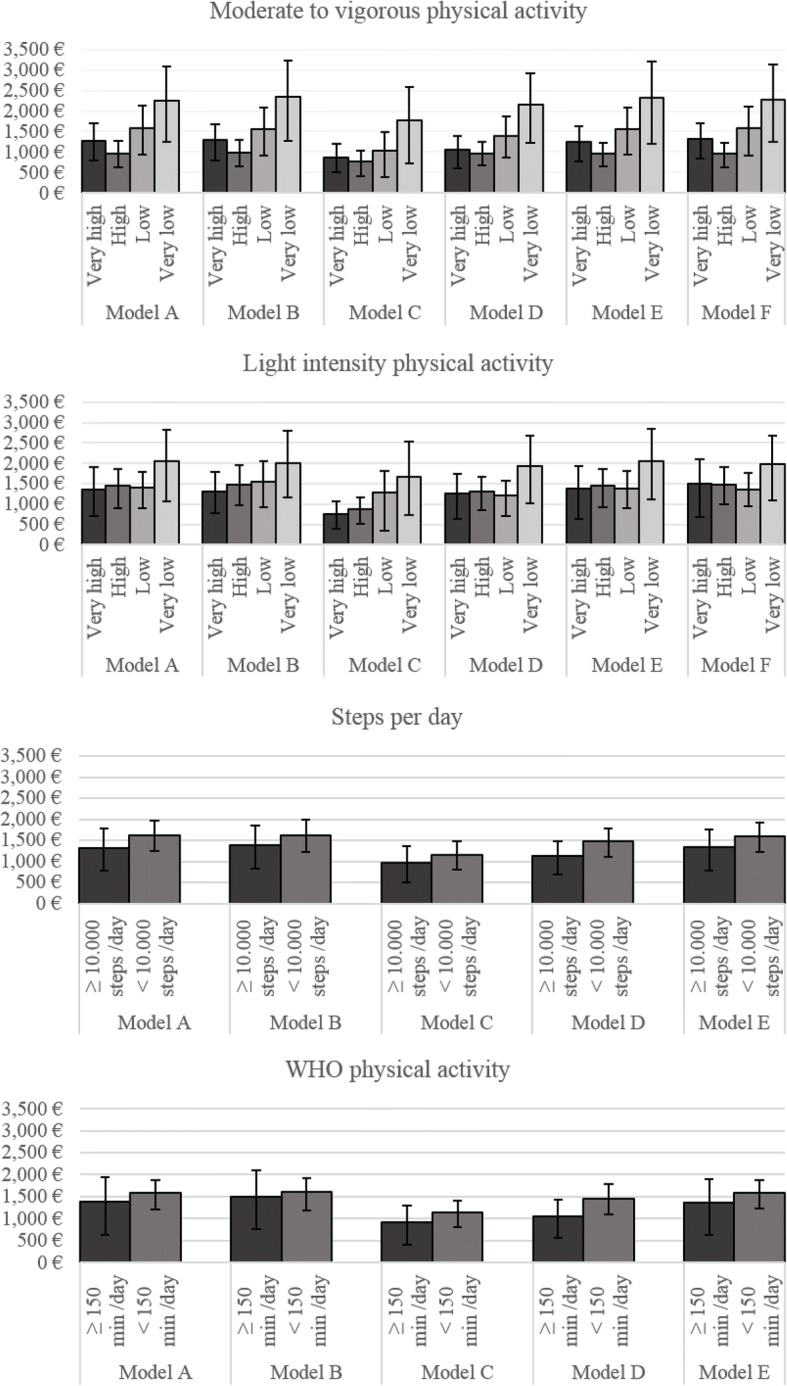
Mean overall direct healthcare costs of device assessed physical activity groups in the subsample - Results from a two-part gamma regression model

Table 4 shows the results of the two-part gamma regression model for the subsample with accelerometer data where subjects with the highest activity were the reference group and excess costs are reported for subjects in the lower activity groups. An overview on overall direct costs for MVPA and light intensity PA groups that resulted from the different models is shown in Fig. 2.

**Table 4:** Overall direct costs (€) associated with PIA estimated with the different models in the subsample

Variable	MVPA (Quartiles); mean and mean difference [95% CI]				Light intensity physical activity (Quartiles); mean and mean difference [95% CI]				Step counts ^g^; mean and mean difference [95% CI]		WHO threshold^h^; mean and mean difference [95% CI]	
	*Very high (Ref.)*	*High* (*β*)	*Low* (*β*)	*Very low* (*β*)	*Very high (Ref.)*	*High* (*β*)	*Low* (*β*)	*Very low* (*β*)	Active *(Ref.)*	Inactive (*β*)	Active *(Ref.)*	Inactive (*β*)
Model A^a^	1276 [841, 1759]	− 308 [− 883, 233]	300 [− 363, 992]	986 [15, 1982]**	1361 [806, 2005]	86 [− 656, 788]	37 [− 774, 799]	685 [− 294, 1838]	1309 [846, 1830]	301 [− 331, 851]	1397 [843, 2175]	186 [− 585, 823]
Model B^b^	1297 [903, 1790]	−304 [− 870, 237]	255 [− 407, 912]	1049 [1, 2278]**	1306 [822, 1834]	181 [− 475, 851]	242 [− 596, 946]	702 [− 242, 1776]	1389 [918, 1955]	239 [− 415, 786]	1498 [901, 2226]	101 [− 684, 704]
Model C^c^	877 [556, 1249]	− 116 [− 606, 362]	149 [− 465, 861]	889 [− 5, 1870]*	765 [465, 1133]	109 [− 355, 560]	518 [− 119, 1287]	912 [29, 1986]**	976 [592, 1459]	167 [− 413, 689]	911 [528, 1423]	235 [− 394, 772]
Model D^d^	1055 [721, 1506]	−91 [− 561, 389]	338 [− 274, 949]	1107 [151, 2166]**	1247 [743, 1849]	53 [−684, 719]	−35 [− 720, 669]	681 [− 246, 1709]	1118 [764, 1551]	358 [−175, 839]	1060 [682, 1547]	401 [− 172, 923]
Model E^e^	1253 [876, 1737]	− 292 [− 246, 797]	310 [− 384, 1027]	1080 [74, 2207]**	1387 [848, 2134]	−56 [− 797, 755]	−5 [− 865, 737]	675 [− 423, 1808]	1331 [904, 1878]	262 [− 354, 878]	1357 [822, 2089]	229 [− 541, 837]
Model F^f^	1315 [927, 1797]	− 351 [− 833, 220]	262 [− 492, 1010]	966 [13, 2097]**	1511 [912, 2349]	−40 [− 987, 694]	− 159 [− 932, 591]	462 [− 619, 1486]	–	–	–	–

The highest level of PA was used as a reference within all four different measures of PA. Therefore, the values in the respective columns (*Ref.)* represents a mean estimate whereas the values in the other columns (*β*) represent coefficients

^a^Model A: adjusted for age, age^2^, sex, education and equivalence income;

^b^Model B: adjusted for variables in Model A plus diabetes, asthma, MI, stroke, cancer, smoking status and alcohol consumption;

^c^Model C: adjusted for variables in Model A & exclusion of participants with prevalent chronic diseases mentioned in Model B (*n* = 1608);

^d^Model D: adjusted for variables in Model A & exclusion of participants with problems with walking (*n* = 2059);

^f^Model F: adjusted for variables in Model A & MVPA or light intensity physical activity, respectively;

^g^active: > 10.000 steps per day, inactive: ≤ 10.000 steps per day;

^h^active: ≥ 150 min MVPA per week in ≥10-min bouts, inactive < 150 min MVPA per week in ≥10-min bouts; ** *p* < 0.05, * trend with *p* < 0.10

#### Device assessed MVPA

In this domain, results showed consistently that participants with very low MVPA had higher direct healthcare costs. When adjusted for age, age^2^, sex, education and equivalence income (*Model A*) the excess costs of participants with very low MVPA overall direct healthcare costs were €986 [15, 1982] in reference to participants with very high MVPA [€1276, 95% CI: 841, 1759]. The results were similar when adding wear time (Model E) or light intensity PA (Model F) as covariates.

#### Device assessed light intensity PA

The association with light intensity PA was qualitatively similar to the association with MVPA, with people with very low light intensity PA having the highest direct healthcare costs. However, this association was only significant for Model C, where we excluded participants with chronic conditions [€912, 95% CI: 29, 1986].

#### Device assessed steps per day

Concerning steps per day (more than or less than 10,000 steps per day), inactive participants had higher direct healthcare costs as compared to active subjects (Model A). However, this difference was not statistically significant.

#### Device assessed WHO PA threshold

The direct healthcare costs of those participants, who achieved the WHO recommendation [1], i.e. those who reached at least 150 min of MVPA in ≥10-min bouts, were not significantly different, but always higher, to the ones that did not reach the WHO threshold.

## Discussion

This study aimed to analyze the association between device assessed and self-reported PA and direct healthcare costs in a population-based sample. Device assessed but not self-reported PA was associated with lower direct healthcare costs. The size of the effect was dependent on the type of model used to calculate the costs attributable to PIA.

It is difficult to compare the findings of this study with other studies assessing PIA and direct healthcare costs because of the numerous different methods that have been used to calculate direct healthcare costs and to assess or estimate PIA [4, 5, 11, 28]. Still, this study is partly in line with other international studies that report higher direct healthcare expenditures for inactive as compared to active subjects [11, 28]. For example, Carlson et al. [11] also investigated self-reported PA and direct healthcare expenditures in the U.S. In this study, PA was assessed by asking adults how often and, if applicable, how long during leisure-time they participated in vigorous-intensity activities and in light- or moderate-intensity activities of at least 10 min duration. Respondents were then classified into three activity levels: active, with 150 min/week of moderate-intensity equivalent physical activity, as recommended by the WHO [1], insufficiently active, i.e. those below this threshold, and inactive, reporting no moderate-intensity equivalent that lasted at least 10 min. Carlson et al. [11] also investigated the relation between PA and direct healthcare costs in a population aged 21 years or older (49.8% females). More than one-third of the participants reported being inactive, compared to 28.3% participants in our study reported to be inactive. Concerning the accelerometer data, the low adherence of this study population to the WHO PA recommendations that has been described elsewhere by Luzak et al. [18] is comparable to other European populations.

Another important issue of interest we tried to address was the various sources of confounding in the relationship between PIA and direct healthcare costs. Our assumption is that inactive subjects cause more direct healthcare costs than active participants, but we cannot exclude the fact that there might be participants who are inactive due to a sickness or disability (exposure) that influence their PA (outcome). One way to address this issue is to exclude participants who report having problems with walking or being unable to do PA [11]. Therefore, we excluded participants who reported walking problems in *Model D*. In *Model D* the observed difference in direct healthcare costs was not statistically significant but still present.

Another way to address the confounding problem would be to use longitudinal panel data. By having information about the timely order of PA and direct healthcare costs it would be easier to disentangle the direction of the association and to control for confounding factors. Ideally, for this one would combine representative population-based data from various follow up examinations with health insurance claims data that provide granular information on the trend of direct healthcare costs over time.

### Strengths and limitations

This paper has several limitations. First, the present results, including subjects of KORA FF4 with a response of 68% limiting the general external validity. Second, our results are based on cross-sectional data, which does not give information about long-term PIA behavior or change of PIA during the time that plays an important role in relation to direct healthcare costs.

In addition, this study did not include indirect costs, which covers loss of productivity from premature death or work absenteeism. The authors are also aware that a constant utilization of health services was assumed in the present study when reported utilizations were extrapolated to a one-year period, which tends to underestimate the actual utilization over one year [29].

A major strength of this study is, that it provides insight into the relationship between self-reported and device assessed PIA and direct healthcare costs and that it is based on a relatively large population-based sample. Another strength of this study is the estimation of various alternative models to assess the robustness of the results with regard to confounding factors.

## Conclusions

The results indicate that device assessed but not self-reported PIA is associated with higher direct healthcare costs. Larger studies with device assessed PA and longitudinal design are needed to better quantify the impact of PIA on direct healthcare costs.

## Additional files

Additional file 1: Matrix-transformation for self-reported sports physical activity. The matrix shows how the participants’ responses regarding their physical activity during summer and winter (1–4) were combined and categorized (I-IV). (DOCX 14 kb)

Additional file 2: Cost calculation for physician visits. The table shows the German unit costs according to Bock et al. [23]. (DOCX 12 kb)

Additional file 3: Physical activity and direct healthcare costs from a cross-sectional perspective. The figure shows possible confounders and mediators for the association between physical inactivity and direct healthcare costs. (DOCX 28 kb)

## Abbreviations

AG MEG: Working Group Methods in Health Economic Evaluation
BMI: Body-Mass-Index
CI: Confidence Interval
DDD: Defined daily dose
IV: Instrumental variable
KORA: Cooperative Health Research in the Region of Augsburg
MI: Myocardial infarction
MVPA: Moderate-vigorous physical activity
PA: Physical activity
PIA: Physical inactivity
WHO: World Health Organization
