# Supplementary material for: Direct healthcare costs associated with device assessed and self-reported physical activity: results from a cross-sectional population-based study
Source: BMC Public Health. 2018 Aug 3;18:966. doi: 10.1186/s12889-018-5906-7 (PMC6090754; doi:10.1186/s12889-018-5906-7)
Supplement: Supplementary file 1 — Matrix-transformation for self-reported sports physical activity. The matrix shows how the participants’ responses regarding their physical activity during summer and winter (1–4) were combined and categorized (I-IV). (DOCX 14 kb) [file 12889_2018_5906_MOESM1_ESM.docx]

|  |  | **PA during summer** | | | |
| --- | --- | --- | --- | --- | --- |
| **PA during winter** |  | 1 | 2 | 3 | 4 |
|  | 1 | **I** |  |  |  |
|  | 2 |  | **II** | **III** |  |
|  | 3 |  | **III** | **IV** |  |
|  | 4 |  |  |  |  |
| 1 “regularly more than two hours per week” I “high activity“  2 “regularly one to two hours per week” II “moderate activity“  3 “less than one hour per week” III „low activity“  4 “no activity” IV „no activity“ | | | | | |
