# Supplementary material for: Direct healthcare costs associated with device assessed and self-reported physical activity: results from a cross-sectional population-based study
Source: BMC Public Health. 2018 Aug 3;18:966. doi: 10.1186/s12889-018-5906-7 (PMC6090754; doi:10.1186/s12889-018-5906-7)
Supplement: Supplementary file 2 — Cost calculation for physician visits. The table shows the German unit costs according to Bock et al. [23]. (DOCX 12 kb) [file 12889_2018_5906_MOESM2_ESM.docx]

|  | **Average visits in 3 months (means)** | **Unit costs per visit for 2013 (in Euro)** |
| --- | --- | --- |
| General practitioner | 2.039 | 20.06 |
| General practitioner (internal medicine) | 1.767 | 20.06 |
| Specialist for internal medicine | 1.688 | 65.44 |
| Gynecologist | 1.102 | 30.13 |
| Ophthalmologist | 1.323 | 34.78 |
| Orthopedist | 2.008 | 25.42 |
| Otolaryngologist | 1.600 | 26.40 |
| Surgeon | 2.1093 | 43.39 |
| Dermatologist | 1.612 | 18.89 |
| Radiologist | 1.460 | 40.06 |
| Urologist | 1.217 | 24.70 |
| Psychiatrist/ Neurologist | 1.488 | 44.72 |
| Psychotherapist | 5.089 | 78.08 |
| Occupational medicine | 1.057 | 40.06 |
| Other physician | 2.171 | 40.06 |
