# Supplementary material for: Direct healthcare costs associated with device assessed and self-reported physical activity: results from a cross-sectional population-based study
Source: BMC Public Health. 2018 Aug 3;18:966. doi: 10.1186/s12889-018-5906-7 (PMC6090754; doi:10.1186/s12889-018-5906-7)
Supplement: Supplementary file 3 — Physical activity and direct healthcare costs from a cross-sectional perspective. The figure shows possible confounders and mediators for the association between physical inactivity and direct healthcare costs. (DOCX 28 kb) [file 12889_2018_5906_MOESM3_ESM.docx]

**Mediator**

- Blood pressure & other medical parameter
- Health status (physical & mental)
- Obesity
- BMI/ WtHR
- occupation

**Confounder**

- Age & Gender
- Sedentary lifestyle
- Educational level/ marital status/ occupation
- Race/ ethnicity
- Problems with walking
- Previous health status
- Genetic profile
- Environment (health policies, healthcare system)
- Health status (physical & mental)
- Obesity
- BMI/ WtHR
- Risk attitude
- Risk behaviour (alcohol/smoking)
